# Local Use of Iodine

**Published:** 1855-11

**Authors:** 


Local Use of Iodine.—Dr. Mikschik states that he has derived great advantage from the tincture of iodine as an emenagogue, applying it externally to the os uteri. Cases which had long resisted all other means, have yielded to the application after the third day. He has not found the advantages that Boinet says attends its use in vaginal catarrh, and from the temporary irritation it produces, it should be avoided in pregnancy, and when the uterus is inflamed. As a means of dispersing organic exudations he much prefers lotions (iod. potass. ℈j., iodin. gr. x., aquæ ℔j.) to ointments. They should be applied on compresses which are to be covered by a cataplsam.—(*Wein Wochenscrift.* 1855. No. 22.) *Ibid.*

